# Reducing antimicrobial use in chicken production in Vietnam: Exploring the systemic dimension of change

**DOI:** 10.1371/journal.pone.0290296

**Published:** 2023-09-08

**Authors:** Chloé Bâtie, Hang Tran Minh, Van Anh Thi Vu, Duong Thuy Luong, Trang Thi Pham, Nicolas Fortané, Phuc Pham Duc, Flavie Luce Goutard

**Affiliations:** 1 ASTRE, CIRAD, INRAE, Univ Montpellier, Montpellier, France; 2 Institute of Anthropology, Vietnam Academy of Social Sciences, Hanoi, Vietnam; 3 USAID Learns, United States Agency for International Development/Vietnam, Hanoi, Vietnam; 4 Institute of Regional Sustainable Development, Vietnam Academy of Social, Hanoi, Vietnam; 5 Faculty of Animal Science and Veterinary Medicine, Thai Nguyen University of Agriculture and Forestry, Thai Nguyen, Vietnam; 6 UMR IRISSO, CNRS, INRAE, Université Paris Dauphine, PSL, Paris, France; 7 Center for Public Health and Ecosystem Research (CENPHER), Hanoi University of Public Health, Hanoi, Vietnam; 8 Institute of Environmental Health and Sustainable Development, Hanoi, Vietnam; 9 ASTRE, CIRAD, Hanoi, Vietnam; 10 National Institute of Animal Science, Hanoi, Vietnam; 11 National Institute of Veterinary Research, Hanoi, Vietnam; UGHE: University of Global Health Equity, RWANDA

## Abstract

Antibiotic use in livestock production is one of the drivers of antibiotic resistance and a shift towards better and reduced antibiotic usage is urgently required. In Vietnam, where there are frequent reports of the misuse and overuse of antibiotics, little attention has been paid to farmers who have successfully changed their practices. This qualitative study aims to understand the transition process of Vietnamese chicken farmers toward reduced antibiotic usage. We conducted semi-structured interviews with 18 chicken farmers, 13 drug sellers, and 5 traders using participatory tools and a socio-anthropological approach. We explored the farmers’ histories, current and past antibiotic usage, methods used to reduce antibiotic use, and motivations and barriers to changing practices. Through the thematic analysis of the farmers’ transcripts, we identified technical, economic, and social factors that influence change. Out of eighteen farmers, we identified ten farmers who had already reduced antibiotic usage. The main motivations included producing quality chickens (tasty and safe) while reducing farm expenditures. Barriers were related to poor biosecurity in the area, market failures, and the farmers’ lack of knowledge. Innovation led to overcome these obstacles included the local development of handmade probiotics and the organization of farmer cooperatives to overcome economic difficulties and guarantee product outlets. Knowledge was increased by workshops organized at the communal level and the influence of competent veterinarians in the area. We showed that the transition process was influenced by several components of the system rather than by any individual alone. Our study demonstrated that local initiatives to reduce antibiotic use in Vietnamese chicken production do exist. As changes depend on the system in which stakeholders are embedded, systemic lock-ins must be removed to allow practices to change. The promotion of locally-developed solutions should be further encouraged.

## Introduction

Reducing antibiotic use (ABU) in the animal and human sectors is critical to fighting the burden of antibiotic resistance (ABR). The veterinary sector has been identified as a major consumer, accounting for nearly 73% of all antibiotics sold worldwide. These antibiotics are used in livestock production for a variety of purposes [1] including treatment, prevention and, in some countries, growth promotion [2]. The usage of antibiotics for prophylactic purposes and growth promotion are key determinants of ABR [3]. Growth promoters were therefore banned in Europe in 2006, resulting in a decrease in antibiotic use [4]. Subsequently, additional measures, such as the implementation of action plans like the “Yellow Card Scheme” in Denmark [5] and the Ecoantibio plan in France [6], have contributed to reducing ABU in Europe. While Oceania, North America, and Europe are expected to contribute the least to the increase in antibiotic sales, Asia is expected to fuel the increase by 10.3% between 2017 and 2030, thus maintaining its position as the highest consuming continent [7]. South East Asia, in particular, is regarded as a hotspot for ABR [8]. Indeed, this region is characterized by countries with growing economies, which leads to a higher demand for meat, leading to a shift toward more intensive production [9]. As intensive production in middle-income countries has been shown to account for 1/3 of the increase in global antibiotic consumption [10], economic and demographic growth have been identified as ABR drivers. Other drivers include the misuse and overuse of antibiotics in the livestock sector, easy access to drugs and the poor quality of drugs, among others [9].

In Vietnam, with a population approaching 100 million, the animal sector accounts for 71.7% of total ABU [11]. However, the livestock production sector is currently undergoing numerous changes. First, in 2018, in response to international demand and following the implementation of the first national action plan to combat ABR in livestock in Vietnam [12], antibiotics were banned for growth promotion usage. Additionally, new regulations were implemented in 2020 to gradually decrease the usage of antibiotics in feed for prophylaxis [13] and to make prescriptions mandatory for the purchase of drugs [14]. Second, the government’s plan is to encourage larger-scale farms and intensify production [15] in a country where most farms are still small-scale or backyard [16].

Chicken meat consumption is now considered the second largest source of animal protein after pork and the number of heads has rapidly increased in the last decades in Vietnam [17]. In particular, since the African Swine Fever (ASF) epidemic, there has been a shift in production from pigs to chickens [18]. According to a recent study, antibiotic sales in veterinary drug shops in two provinces in northern and southern Vietnam, were highest in poultry production, as compared to pigs and cattle [19]. The usage of antibiotics in chicken production in Vietnam has been widely documented. Antibiotics (AB) are commonly used to treat and prevent diseases in livestock and are readily available over the counter at affordable prices [20–22]. Moreover, chicken farmers rely heavily on their own experience when using drugs, leading to the overuse and misuse of antibiotics [16, 23, 24]. Three production systems can be identified in Vietnam with different ABU practices [16, 25, 26]. Integrated farms rely on the advice of the integrator, family commercial farms rely on their own experience and household farms rely on the advice of drug sellers (Box 1). In this study, family commercial farms are identified as overusing AB [16]. In another recent study, stakeholders of the veterinary drug value chain identified small-scale family commercial farms and household farms as major barriers to the implementation of new regulations to reduce ABU. They were described as being solely focused on profit, lacking knowledge, strongly attached to the habit of using antibiotics, unwilling to change, and too numerous to be controlled (unpublished data). However, an intervention study has successfully demonstrated that with good veterinary advice, farmers were able to change their practices and reduce ABU [27]. Further studies are required in this direction to identify levers and barriers for farmers in order to lead a change in practices in Vietnam.

**Box 1. Chicken production systems in Vietnam [16]**

- Integrated farms: farmers have a contract with an integrator that provides them with feed, day old chicks (DOCs), drugs, and technical advice; in return the farmers raise chickens for a salary defined according to the Feed Conversion Ratio (FCR) and mortality rate. Exotic breed chickens are sold to supermarkets and cross-breed chickens to traditional markets.
- Family commercial farms: independent farmers who buy feed, drugs, DOCs, and receive technical advice from level 1 or 2 agencies (drugstores), or drug and feed companies for larger scale farms. Chickens are mostly sold to traditional markets through traders and sometimes to supermarkets or other shops specialized in quality chickens.
- Household farms: independent farmers who raise a small number of chickens and combine several activities. Feed and DOCs are usually on-farm products, drugs and technical advice are received from level 2 agencies. Chickens are mostly self-consumed or sold to local markets or for traditional events.

To understand the transition process of Vietnamese chicken farmers toward antibiotic use reduction, we conducted a qualitative study using participatory approaches. Participation is a way to involve animal health stakeholders in the identification of farm management and health-related problems to develop more suitable solutions [28]. Qualitative research allows a deeper exploration of the complex social interactions among individuals within the system in which they are embedded. It allows us to explore different points of view and opinions related to a problem. Rather than focusing our approach on individuals alone, we aimed to explore the systemic factors that influence changes in practices. Indeed, the decision to implement change does not depend solely on a rational decision based on financial considerations but depends also on the social context of farmers [29]. Recent studies have highlighted the need to study ABU and ABR though a systemic perspective rather than focusing on better knowledge, as argued by knowledge deficit models [30]. For this purpose, we used the concept of “trajectory of change” that derives from the multi-level perspectives of transition theory [31]. This concept has been used to understand farmer transition pathways toward the reduction and better use of antibiotics in pig production [32] and chicken production [33] in France. It is based on the idea that change is a long-term process [34] that depends on the social, technical, and economic factors of the system within which the stakeholders are integrated [35]. The goal of the present study is to shed light on the transition process of Vietnamese chicken farmers toward a reduced antibiotic use. We employed a systemic approach to this issue, to identify the factors that influence farmer transition pathways and whether local on-farm innovations were developed to adapt to the changes. Through this study, we aim to contribute to more targeted recommendations by understanding the system in which farmers are embedded and identifying levers within this system that can be activated.

## Methods

### Study zone and selection of the participants

Our study was conducted in the Phu Binh district in Thai Nguyen province in the midland and mountain region of northern Vietnam. Thai Nguyen province is located 70km north of Hanoi, the capital of the country. Thai Nguyen was selected because of its large poultry production (15 407 million heads in 2020) [17], its diversity of chicken production systems, its proximity to Hanoi, and the existing collaboration with local partners. According to the Sub-department of Animal Health and Livestock Production (SubDAHLP) that represents the veterinary services at the provincial level, it is a leading province for livestock production and emblematic for what is likely to happen in other provinces. Together with SubDAHLP and the Thai Nguyen University of Agriculture and Forestry (TUAF), the district of Phu Binh was selected because of its size, the accessibility of the farms, the openness of breeders to share their experience, and information about the district.

The study was designed in two phases. Phase 1 aimed to gather information on the different production systems in the district, on antibiotic usage, and to identify farms with lower ABU. The aim of phase 2 was to explore the network of respondents who have reduced their antibiotic usage, identified in phase 1. In phase 1, 10 farmers and 10 drug sellers were selected by the agriculture service center of Phu Binh district and TUAF team members based on pre-defined criteria to maximize the diversity of participants. Farmers were selected according to the production system as defined by [16]: integrated farms, large-family commercial farms, small to medium-family commercial farms, and household farms. Drug sellers were selected according to their structural position in the veterinary drug value chain: communal veterinarians, agencies selling drugs, agencies selling feed and drugs, and independent veterinarians (unpublished data). Based on the interviews conducted in phase 1, additional farmers and drug sellers were recruited for phase 2, following information from local authorities and using the snowball sampling method. Interviews with traders working in the same district were also conducted to explore the influence of the market on changes in practice.

### Data collection

In-depth, face-to-face interviews were conducted in December 2021 (phase 1) and February 2022 (phase 2) by the first author and a team of researchers composed of one researcher from TUAF, one from the Institute of Anthropology of Hanoi, two free-lance researchers in sociology and a variable number of students from TUAF depending on the phase. The translation was done by a student and one sociology researcher for phase 1 and another sociology researcher for phase 2. Members of the team were trained on research objectives, data collection methods, and participatory epidemiology. Before the data collection, a pilot study in Phu Binh district was organized in November 2021 with one farmer and one drug seller. As there were few changes made to the interview guide and considering the importance of the information collected, this farmer’s interview was included in the study.

**Fig 1:**
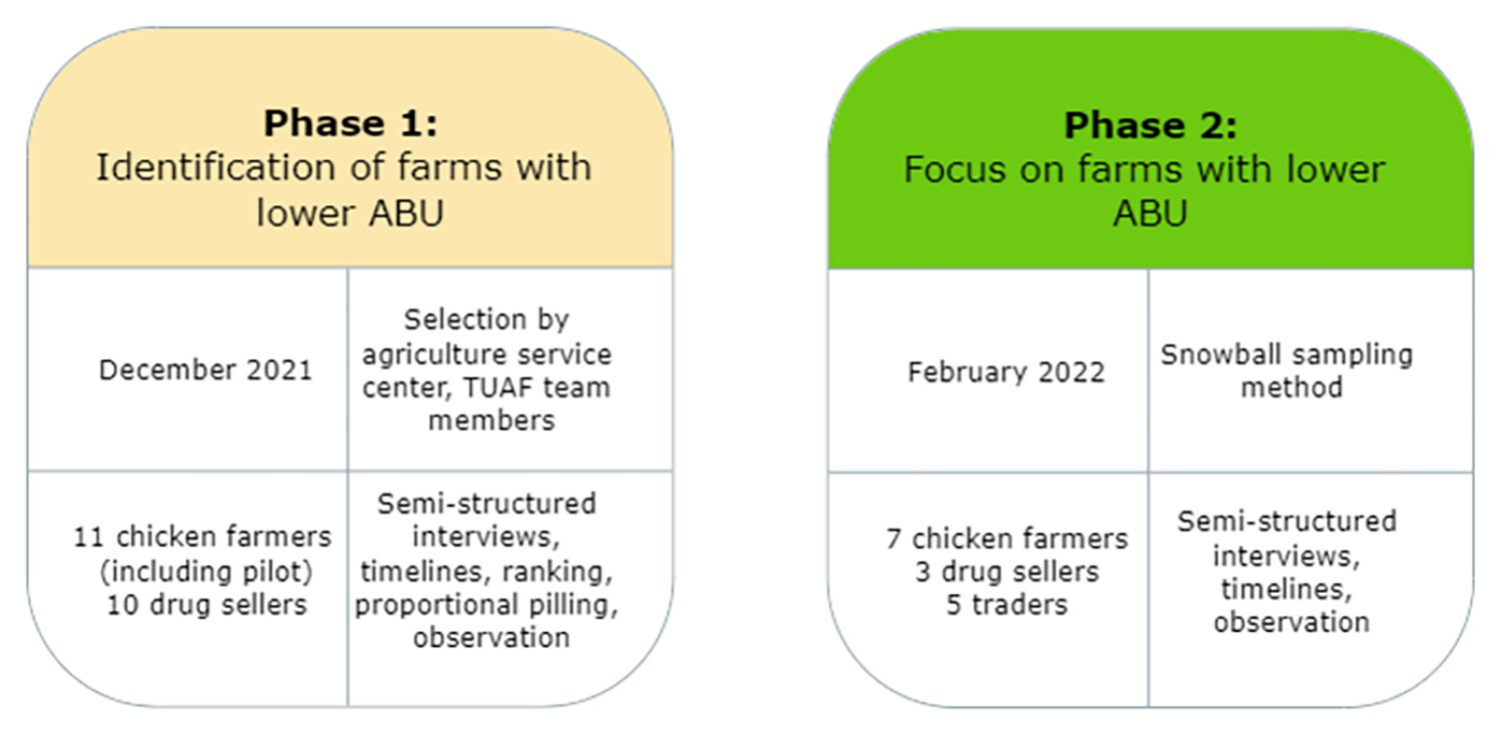
Summary of the data collection method for phase 1 and phase 2 in Phu Binh district, Vietnam, 2021–2022. Top left: dates of the data collection: top right: sampling methods; bottom left: number of participants; bottom right: method of data collection.

The interview guide incorporated participatory epidemiology tools [36] and socio-anthropological approaches. It allowed us to understand why farmers behave the way they do and to explore the type of interaction between the different participants. These participatory approaches helped us to increase the involvement of respondents during the data collection process. The interview guide was tailored to the respondent’s profession (farmer, drug seller, or trader) and organized around four themes: 1) history of the farm/shop and the respondents; 2) technical characteristics (value chain, farm management, AB practice); 3) opinion on antibiotic use and antibiotic resistance, evolution of ABU; 4) antibiotic use reduction (barriers, motivations, impacts, solutions) (S2 File). We used a visualization method (timeline) and ranking and scoring methods (simple ranking and proportional pilling) [36]. First, to explore the evolution of the farms over time, we asked the respondents to draw a timeline of the different events that occurred at the farm level. Then, to explore the different methods used to reduce ABU, we asked the respondents to enumerate them and we wrote them on a card. Then we asked the respondents to rank them according to their perceived effectiveness and feasibility. Finally, to assess the evolution of the farm’s expenses over time and the consequences of ABU reduction on them, we used the proportional pilling tools. Farmers listed their farm expenses and were then asked to divide 100 beans according to the percentage of expenses in each category at the time of the interview. The same procedure was conducted for their expenses five years ago. The timeline of the farm events, ranking of the methods to reduce ABU, and the proportional pilling on farm expenditure were only done for phase 1. Methods during the second phase were only mentioned but not ranked. The interviews were completed by on-farm observation of feeding, housing, herding activities and drug packaging. With the drug sellers, we observed the different products sold in the shop and the interactions between the participants and other sellers or buyers. During phase 1 and phase 2, we stayed on two different farms that were interviewed during the data collection period to observe farm activities over several days (Fig 1).

The study’s objectives were explained to the participants by the interviewers and written consent was obtained before starting the interview. The anonymity of the participants was guaranteed throughout the study. This study was approved by the Ethics Review Board for biomedical research of Hanoi University of Public Health with the application number 021-391/DD-YTCC. The permit to conduct this work was given by the Sub-department of Animal Health and Livestock Production (SubDAHLP) of Thai Nguyen province.

### Inclusivity in global research

Additional information regarding the ethical, cultural, and scientific considerations specific to inclusivity in global research is included in the S1 File.

### Data analysis

Interviews were recorded, fully transcribed in Vietnamese, translated into English by a professional and compiled with field notes. Data from farmers’ transcripts were analyzed by thematic analysis using the six-step method explained by Castleberry and Nolen [37]. Thematic analysis is an inductive approach, developed from grounded theory, that allows meaning to emerge from the data itself. The coding was performed on NVivo software (V.12,2, QSR International). The grid was first constructed *a priori*, according to the interview guide, and validated by the team. All the transcripts were first read in full, coded by the first author and the grid was refined during the analysis. When no new themes emerged from the transcripts, the codebook was validated after team discussion. The codes were analyzed together in light of the research questions and the context of the study to generate themes using thematic hierarchies. We used a backward and forward approach to data coding, the theme emergence, and analysis [37]. The transcripts of drug sellers and traders were not analyzed through thematic analysis but the information was used to explain some of the findings of the farmers’ transcripts analysis.

## Results

### Respondents’ characteristics

Eighteen farmers were interviewed, one during the pilot study, ten during the first phase, and seven during the second phase. Three farmers were under contract with an integrator and raised hybrid or exotic chickens in confined housing with high levels of biosecurity in an intensive system, hereafter referred to as integrated farms. One farmer kept a small number of local breed chickens for his consumption, this is subsequently referred to as the household farm. Fourteen farmers raised free-range local chickens in a semi-confined system, these are later called family commercial farms. Four farmers were part of the same cooperatives (farmers 9, 11, 13, 17) and two farmers wanted to develop a cooperative together (farmers 3,4).

These interviews were completed by the interviews of 13 drug sellers, 11 in the first phase and 2 in the second phase. One drug seller was removed from the data analysis due to irrelevant and incomplete answers. Two drug sellers worked for a drug company (one was also a vaccine company and the other specialized in alternative feed additives), two were communal veterinarians (who were also private veterinarians), one worked for the agricultural service center, five were the owners of a level 1 drug agency, and three were the owner of a level 2 drug agency. Two inter-provincial traders and three traders for small-scale farms were also interviewed in the second phase. One farmer and one drug seller were interviewed twice during each phase because they were considered as key stakeholders and that further investigation in their role was required. Most of the respondents were male, the farmers were above 40 years of age while the drug sellers were between 25 and 40 years old. The education level of dug sellers was higher than that of the farmers. Most of the interviews were conducted in Tan Khanh commune or the neighboring commune. The socio-demographic characteristics of the respondents are summarized in Table 1. The characteristics of the farmers and their farm history are summarized in Table 2, the full table is presented in S1 Table.

**Table 1:** Sociodemographic characteristics of farmers, drug sellers, and traders interviewed during semi-structured interviews in December 2021 (phase 1) and February 2022 (phase 2) in Phu Binh district, Thai Nguyen province, Vietnam.

		*Farmer*	*Drug seller*	*Trader*
**Number**				
Phase 1		10	11	0
Phase 2		8	2	5
Total		18	13	5
**Gender**				
Male		15	9	2
Female		3	4	3
**Age**				
<25		1	0	*NA*
25–40		4	9	1
40–65		13	4	1
>65		0	0	*NA*
**Education**				
Primary		2	0	*NA*
Secondary		4	0	1
Highschool		7	2	*NA*
College		3	4	*NA*
University		2	7	1
**Years of experience**				
<5		2	2	*NA*
5–10		3	5	*NA*
11–15		6	3	2
>15		7	3	*NA*
**Commune**				
Tan Khanh		13	4	3
Bao Ly		0	3	0
Other		5	6	2
**Production system**				
Integrated		3		
Family commercial	Large	4		
	Medium	5		
	Small	4		
Household		1		
**Category of drug seller**				
Agency 1			5	
Agency 2			3	
Communal vet.			2	
Independent vet.			1	
Drug company			2	

Integrated farm: farm in contract with an integrator; family commercial farm: independent farm raising chickens for commercial purposes; household: few free-range chickens for own consumption (Bâtie et al., 2022a); Large: >5000 chickens/flock, medium: 2000–5000 chickens/flocks, small: 100–2000 chickens/flock; Agency 1: drugstore supplied directly by drug companies and supplying farmers and agencies 2; Agency 2: drugstore supplied directly by agencies 1 and supplying small-scale farms in majority (Bâtie et al., 2022b); NA: not addressed.

**Table 2:** Farmer’s characteristics according to semi-structured interviews from December 2021 (phase 1) and February 2022 (phase 2) in Phu Binh district, Thai Nguyen province, Vietnam.

Farmer (phase of interview)	Gender, age, education	Years of experience	Date of installation	Production system, number/flock, type, housing, number of workers	Description and farm evolution	ABU profile
**Farmer 0 (pilot)**	Male, 48, primary school	15	2008	Family commercial, 15000, DOCs, semi-confined, 1	Independent farmer since 2017, main activity	Have reduced
**Farmer 1 (1)**	Male, 25, university	3	2021	Contract, 15000, meat, confined, 4	Farm manager, change company in 2021, main activity	Have reduced
**Farmer 2 (1)**	Male, 32, university	5	2021	Contract, 40000, meat, confined, 4	Farm manager, owner of another farm of the same chicken company, main activity, previous job at the veterinarian faculty	Have reduced
**Farmer 3 (1)**	Male, 45, college	20	2015	Family commercial, 4000, meat, semi-confined, 2 (family)	Farm owner, main activity, want to develop a cooperative	In the process of reducing, same amount
**Farmer 4 (1)**	Male, 47, highschool	15	2007	Family commercial, 3000, meat, semi-confined, 1	Farm owner, also raising pigs and dogs, want to develop a cooperative	In the process of reducing, increased amount
**Farmer 5 (1)**	Male, 44, highschool	14	2016	Family commercial, 3000, meat, semi-confined, 1	Farm owner, main activity	Increased
**Farmer 6 (1)**	Male, 36, highschool	12	2008	Family commercial, 2000, meat, semi-confined, 1	Farm owner, stop raising pigs (ASF), crops	In the process of reducing (?), same amount
**Farmer 7 (1)**	Female, 45, highschool	12	2013	Family commercial, 1500, meat and eggs, semi-confined, 1	Farm owner, depending on the market conditions switch between laying hens and broilers, multiple activities (drug seller)	In the process of reducing (?), increased
**Farmer 8 (1)**	Male, 42, college	14	2018	Family commercial, 6000, DOCs, semi-confined, 2 (family)	Farm owner, stop raising pigs, main activity	Same amount
**Farmer 9 (1)**	Male, 48, highschool	16	2016	Family commercial, 5000, meat, semi-confined, 2 (family)	Farm owner, husbandry and forestry, member of the cooperative	Have reduced
**Farmer 10 (1)**	Female, 52, secondary school	32	1989	Household, 100, meat, eggs, DOCs, free range, 3 (family)	Farm owner, multiple species, crops	Same amount
**Farmer 11 (2)**	Male, 42, highschool	13	2021	Family commercial, 1000+1200, meat and eggs, semi-confined	Farm owner, main activity, member of the cooperative	Have reduced
**Farmer 12 (2)**	Female, 41, secondary school	19	2012	Family commercial, 1000, meat, semi-confined, 2 (family)	Farm owner, main activity	In the process of reducing (?), same amount
**Farmer 13 (2)**	Male, 37, secondary school	13	2009	Family commercial, 4000, meat, semi-confined, 2 (family)	Farm owner, main activity, member of the cooperative	Have reduced
**Farmer 14 (2)**	Male, 54, primary school	5	2017	Contract, 8000, meat, confined, 1	Farm manager, main activity, backyard flock for own consumption	Have reduced (don’t want to reduce)
**Farmer 15 (2)**	Male, 56, secondary school	22	2000	Family commercial, 1800, meat, semi-confined	Farm owner, buffaloes, stop raising pigs	Have reduced
**Farmer 16 (2)**	Male, 38, highschool	20	2002	Family commercial, 7000, meat, semi-confined, 2 (family)	Farm owner, main activity, VietGAHP certificate	Increased
**Farmer 17 (1,2)**	Male, 58, college	20	2001	Family commercial, 4000, meat, semi-confined, 2 (family)	Farm owner, main activity, leader of the cooperative Leader of the cooperative VietGAHP certificate	Have reduced

AB: antibiotics; ASF: African Swine Ver, VietGAHP: Vietnamese Good Animal Husbandry Practices: (?): unclear.

In the district of Phu Binh, the most common farming system was to raise free-range local breed chickens (*Gà Ta Lò*) with access to a barn for 4 to 5 months (semi-confined housing). They represented 14/18 interviewed farmers. Most of these farmers had benefited from governmental programs to develop a brand of “hill chickens” (Gà đồi) from this area. Thus, most farmers had been raising chickens for a long time and the evolution in their farming practices was mostly related to an increase in flock size and infrastructure that was encouraged by governmental programs. Chicks were usually bought nearby from relatives or other farmers and feed and drugs were also provided locally. Most chickens were purchased by traders through middlemen for sale in the Bac Giang wholesale market and then distributed to Hanoi or Bac Ninh provinces. Moreover, according to the respondents, the number of drug sellers had also increased in the area together with the development of livestock production. Integrators with national or international influence were present in the district but represented a minority. In the case of integrated farms, the integrator provided farmers with feed, drugs, day-old chicks (DOCs), and advice and then collected and distributed the final products. White chickens were usually raised with a shorter production cycle than the local breed (around 55 days) and raised in a confined system with strict biosecurity protocols. Households represented a high proportion of farmers in the district, with local, entirely free-range chickens raised mostly for the family’s consumption as the household feed them with waste food or their agriculture products (paddy, vegetables). Only one household was interviewed as they were identified as low AB users.

### Antibiotic usage

All farmers used antibiotics for the treatment of sick chickens. In the presence of disease, they deem antibiotics to be the only solution and a necessity for animal health welfare. The most common diseases mentioned by farmers and drug sellers were enteritis, typhoid, asthma, coccidiosis, and blackhead disease. Farmers also reported an increase in “*bệnh ghép*” also called “serious disease” by farmers and drug sellers. They perceived it as serious because it was a combination of several diseases and was more complicated to treat. In the case of *bệnh ghép*, they used a combination of several antibiotics, with higher dosages and higher concentrations of AB in the product (called “strong” antibiotic). Antibiotic use for treatment was seasonal, with greater use during the winter, a period particularly suitable for the development of *E*. *coli* and respiratory diseases due to heavy rainfall and cold weather. The dosage and route of injections depended on the severity of the diseases, with the injection identified as the most efficient form for severe disease. Dosage depended on the weight of the chickens, but even if farmers knew that they had to follow product instructions, they increased the dosage of their own accord or upon recommendations made by some drug sellers. They increased the dosage for “serious” diseases because chickens did not eat or drink all the products or because of the poor quality of the drugs. Antibiotics commonly used by farmers were amoxicillin, ampicillin, oxytetracycline, doxycycline, cefotaxime, enrofloxacin, and colistin. Antibiotics were supplemented with alternative feed additives to increase chickens’ resistance and cure them more quickly. Disease diagnostics (clinical examination and autopsy), and treatment choices were usually made by farmers according to their own experience in the case of family commercial and household farms. However, when facing a “serious” disease or unknown clinical signs other farmers were consulted for advice or relied on health care professionals. In this case, treatment choices were made based on the farmer’s description of clinical signs, farm visits, and autopsy.

Of the 18 farmers interviewed, 12 used antibiotics for prevention periodically throughout the animal’s life, during the first week usually to prevent typhoid, or at the change of season when it was going to rain, even though the chickens were vaccinated. In prevention, antibiotics were mainly used to prevent asthma and enteritis. Respondents reported more preventive use given periodically throughout the animal’s life for laying hens than broilers. antibiotics were mostly. In some cases, the AB used were different at each production cycle. The most common form was powder, which was then mixed with the feed. In prevention, they used lower concentrations of AB in the product (“weak” antibiotic), or they used half the dose as indicated on the packaging. Some farmers believed that it was necessary to use AB for preventive purposes, as this would reduce the disease incidence or its severity, that “prevention is better than treatment” and that it would lead to a reduction in costs. This belief was also shared by some drug sellers. No feed containing AB was found during our survey. Independent farmers reported that it was no longer possible to find feed containing antibiotics for adult chickens, and contract farms explained that they only used feed with AB in the early stages of the production.

“*It is used periodically for 3 days/time*. *Sometimes 1 day use 1 day not*, *and sometimes 4–5 days/time*. *It depends*, *if I see chickens are weak*, *I will use 3 days/time*, *if they are good I use 1 day and 1 day don’t use it*. *It depends*.*” (Farmer 5)*

For three farmers it was not clear whether they used antibiotics for prevention or not. Three farmers did not use antibiotics for prevention because, in their opinion, it should not be used at a preventive method but only for treatment. For the same reason and to avoid the overuse of antibiotics, some drug sellers did not recommend the usage of antibiotics for prevention.

Despite the common use of antibiotics, their overuse was also perceived negatively by farmers, drug sellers, and traders. They considered that the overuse of antibiotics would affect the quality of the chicken meat with changes in appearance (spots and rough feathers), unhealthy chickens, and improper growth. And as traders buy chickens based on their appearance, this would result in a loss of income (chicken with visual defects are usually bought 10% cheaper).

### Perception of antibiotic resistance

Farmers were well-acquainted with the requirement to stop using antibiotics before selling chickens. They were aware of the negative consequences for human health due to residues in meat. One farmer who raised household chickens did not want to buy chickens from large farms because of the presence of residues and it was common for traders or consumers to wait a few days before eating the chickens. However, as one respondent reported, the withdrawal time was not always respected in practice. As farmers were using too much antibiotic, it took time to eliminate, and waiting would lead to an increase in expenditure (especially for feed) and a loss of income. This situation was facilitated by the lack of residue control on the traditional market. However, according to one respondent, traders were able to recognize if a chicken had been treated recently with AB. However, integrated farms were subject to random controls by the integrators and thus stopped using antibiotics 35 days prior to selling. The VietGAHP certificate (Vietnamese Good Animal Husbandry Practices) is a voluntary certification that allows products to be sold in supermarkets. Farmers with this certificate were also particularly concerned about respecting this withdrawal period.

“*Some households even though their chickens were injected just 1–2 days before*, *still sell them to the dealer (…)*, *if the middleman knows how to check the flock*, *he will find that the injected area will always be brutally bruised*.*” (Farmer 6)*

Farmers were generally aware of antibiotic resistance, even though the level of knowledge varied between respondents. The most common definition of antibiotic resistance was that the antibiotic did not work and that they had to change antibiotics. A similar definition was provided by drug sellers.

“*We often call this issue “nhờn thuốc” (antibiotic resistance)*, *it means that that medicine doesn’t work although it is very new*, *very recent*, *very good*, *with a high antibiotic content” (Farmer 0)*

ABR was identified as being mainly caused by the overuse or misuse of antibiotics and the continued use of the same antibiotics. Some farmers and drug sellers also believed that the cause was other farmers lack of knowledge on disease diagnosis. Farmers also reported that antibiotic resistance occurred because of the soil pollution. This meant that when several flocks of chickens were raised in the same area, there was higher disease incidence and the presence of antibiotics in the environment. The identified consequences of ABR were that chickens couldn’t be cured, they had to switch to other antibiotics and increase the dosage, and this resulted in decreased productivity. Most farmers had experienced this on their farms. However, few respondents made the link between antibiotic resistance and the risk to human health. Their main source of information about antibiotic resistance was workshops organized by agencies, drug sellers, governmental projects, and TV channel media.

“*The first reason is the increasing population of chickens*. *Secondly*, *it’s because the area of land doesn’t change*, *but I don’t have much time to rest in between different flocks*. *The chickens are not raised in cages*, *but they’re pastured*, *so they would defecate on the land after eating*. *Therefore*, *the land would get polluted*.*” (Farmer 6)*

### Usage and motivations to reduce usage of antibiotics

The evolution over time of antibiotic usage was perceived either as an evolution in price or quantity (supplementary materials for the results of the proportional pilling). Respondents reported an increase in the price of antibiotics and thus in their farm expenditure. The increase in farm expenses was up to twice as high as a few years before, as confirmed by drug sellers. This was also related to higher amounts used (usually twice of the recommended dosage) due to higher disease incidence, intensification of production (overlap between two batches, higher density), and treatment difficulties.

We identified three ABU profiles (Table 2). Three farmers had increased their antibiotic usage and had no plan to reduce it in the near future. Five farmers had not reduced their usage but intended to reduce it. Ten farmers had reduced their antibiotic use, including three integrated farms, four farmers from the same cooperative, two independent farmers, and one household farm. Drug sellers reported an increase in chicken production in the area, with larger scale farms leading to higher AB sales. However, some of their customers were using less antibiotic because they were using more vaccines and applying better farming practices. Motivations and barriers to reducing antibiotics for each respondent are detailed in S1 Table. Integrated farmers were obliged to comply with the requirements of the integrator that sought to sell on the international market, by complying with norms and regulations, or to develop a new domestic market for quality products. One farmer chose a specific integrator because they used less AB. For family commercial farms, reducing AB was often perceived as a way to reduce farm expenditure, improve farming practices, be more productive, increase the quality of the products and produce products that are safer for consumers. The main barriers included the high disease incidence linked to the free-range production system, on-farm density, and high density of the farms in the area. The household farmer had reduced her ABU because she reduced the number of chickens on her farm. Production was then for her own consumption only and she did not want to use AB as it would be harmful to her health.

### Adoption of technical solutions

Farmers adopted technical solutions that contributed to ABU reduction on their farms; these varied according to the participants and their different socioeconomic contexts. Methods for reducing antibiotics were vaccination, alternative feed additives (probiotics, vitamins, electrolytes, tonics), hygiene and sanitation, herbs, and improvement of farming practices. The results of the ranking of these methods by the first phase respondents according to their perceived effectiveness and feasibility are presented in S2 and S3 Tables.

Farmers reported an increasing use of vaccination. The main reasons for this were the intensification of poultry production and the higher disease incidence in the area, related to higher farm density. They also explained that an influential drug seller in the area was promoting good vaccination practices. This drug seller was well-equipped for vaccine storage and farmers trusted in his advice. Moreover, farmers communicated together on the need of using vaccines.

“*We usually say to each other that the chickens live thanks to vaccines*, *they cannot live without vaccines*.*” (Farmer 3)*

Farmers 9, 11, and 13—all members of a cooperative—used local handmade probiotics produced by the cooperative leader (farmer 17). The homemade probiotics were made at the farm-level in a dedicated room from the fermentation of leaves and fruits. It was then mixed with the result of the digestion of cereals by earthworm and flies’ larvae to increase chicken’s growth. They started using these products in line with the technical advice provided by the leader of the cooperative and also because they considered this method to be successful in many aspects. Indeed, probiotics helped to reduce farm expenditures by enhancing chickens’ resistance and thus reducing ABU, it also allowed them to feed the chickens with corn-based feeds that were cheaper than industrial feeds. There was less disease among the chickens and they were of better quality. For example, the farmer who developed these probiotics reported a reduction of 30% in his ABU. One disadvantage of using probiotics was the increased workload due to the need to grind the corn and mix the probiotics manually. To overcome this problem, the leader of the cooperative planned to buy a mixer that other farmers could use.

Using probiotics was also a way to obtain a better valorization of the products. Indeed, a new cooperative was created by farmer 17 where probiotics are a requirement. Chickens are sold with the label “fed with organic probiotics” so that obtain a higher price. A better valorization of products was also the objective of one integrator. Using herbs was thus a marketing strategy for the integrator to produce quality chickens and to move toward the production of AB-free chickens.

Integrated farms also had to follow the strict biosecurity standards of the company (pad cooling, disinfection, automatization, …) and perceived this as highly effective to reduce disease incidence. For family commercial farms, the most common biosecurity measures were hygiene and sanitation (i.e. cleaning and disinfection of the barn) as these were also seen to be a cheap and easy way to reduce the disease incidence. Reducing chicken density, observing a fallowing period and not overlapping flocks were identified as effective means to reduce the use of antibiotics. However, they were not always applied because of the perceived reduced profitability and the need for farmers to increase their volume of sales to maintain a sufficient income.

Several farmers improved their farming practices, usually by attending workshops, and thus reduced their antibiotic usage. They usually wanted to learn more about how to improve farming practices and reduce farm expenditure. One farmer completely changed his feeding and drinking systems to improve farming conditions and provide a safer environment for the chickens (“eat clean, drink clean, and stay clean”) and his ABU has since decreased. Another farmer considered that spending time with the chickens was an efficient way to reduce ABU. Indeed, on his previous farm, he spent limited time observing the chickens. Since he started working on another farm with more human resources, the disease incidence had decreased. Cooper sulfate, bio mattress, lime, and rice husks with probiotics were also used to keep the environment dry.

“*Taking care of chickens in the right way can also help to reduce diseases*. *For example*, *if we don’t take care of them like children*, *they will be weak and thin*. *Conversely*, *if we give thoughtful care to them*, *they will rarely get sick*.*” (Farmer 1)*

### Influence of social networks on reducing antibiotic usage

Social networks supported the diffusion of practices and knowledge between farmers and with health professionals. The impact of social networks on changing practices was higher within the context of a trusted relationship rather than with a seller-buyer one. It also supported mutual aid and the diffusion of solutions. Most farmers participated in several workshops organized by the private and public sectors through the agricultural extension center. In these workshops, stakeholders explained new diseases, diagnostics, and treatments, and introduced new products or vaccines, without specific training on ABR. Usually, products (alternative feed additives) or goodies were distributed to the participants. Another example was the case of a farmer who, after meeting a professor, started to produce his own local handmade probiotics. Several governmental and research projects were also implemented in the area allowing farmers to change their farming practices. For example, one project aimed to increase on-farm biosecurity or to develop VietGAHP farms by providing farmers with technical support and financial assistance. However, the covid pandemic had slowed down the farmers participation of the workshops and on-farm intervention of such projects.

“*Moreover*, *by attending seminars*, *I gained more knowledge about how to use medicine and antibiotics*. *It’s easier when I already have some understanding of it*. *Knowing how to use medicine is a way to prevent drug resistance among chickens*.*” (Farmer 9)*

**Fig 2:**
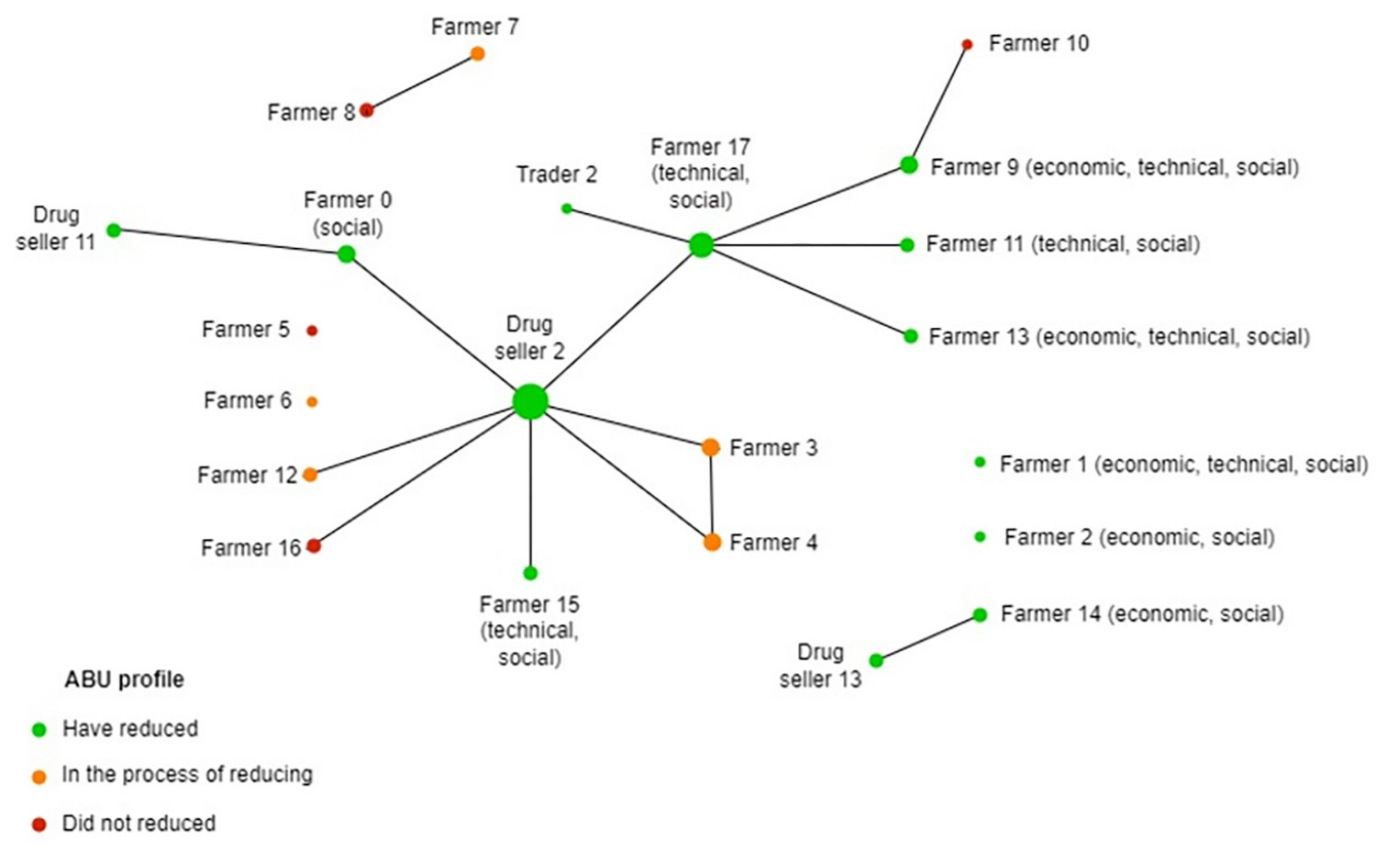
Relationships between the respondents, their antibiotic usage profiles, and factors influencing their changing practices from semi-structured interviews, December 2021 and February 2022, Phu Binh district Vietnam. In brackets: factors influencing change. Circles are proportional to the number of relationships.

Relationships between farmers and drug sellers varied and so did the impact on their antibiotic use. Drug sellers had different backgrounds from high school education, without proper veterinarian training, to universities. To compensate for the difference in knowledge between drug sellers, one veterinarian was training other drug sellers. Most farmers interviewed bought drugs from the same drug seller (DS2 on the Fig 2). This drug seller was also a member of the SubDAHLP staff and was perceived to have a strong influence on the farmers because he had a lot of experience and knowledge. Farmers trusted him and followed his recommendations. In particular, this drug seller was concerned about ABR and ABU and promoted the usage of vaccination. Most farmers did not seek professional advice when they considered that the disease was not serious or for routine antibiotics (i.e. preventive use). They acted on the basis of their own experience. Drug sellers considered farmers to be experienced as they had been raising chickens for a long time. However, farmers asked veterinarians or drug sellers for advice when the diseases are too serious—*bệnh ghép*—because drug sellers know how to mix different drugs. Sometimes, drug companies visited farms directly, or when agents requested it, for difficult cases. Some companies advocated a reduced antibiotic usage and because they specialized in alternative feed additives, they could influence farmers to reduce their antibiotic use. However, most of the drug sellers interviewed did not actively promote a change in practice and it was unclear whether or not farmers would have listened to them. Finally, integrated farms received technical support from the company and were trained from the beginning to raise chickens.

“*[Drug seller 2] has a lot of experience*. *He is loved by so many people (…) [he] would share the experience in dissecting and examining to diagnose the disease in chickens*. *Secondly*, *he would help us in vaccinating when chickens are 1 day old*.*” (Farmer 4)*

The exchange of knowledge among farmers was common, especially because most people had relatives in the area. However, not all of them actively encouraged a change in practice. They would share their experience and farmers would decide whether or not to use the new technics. However, as the interview was conducted across a limited area, most of the farmers were relatives. This was the case of the farmer who started the cooperative and produced probiotics. This made it easier for him to convince the other farmers to try it. He also organized meetings to present his product.

“*[Farmer 17] told me about that*. *He experimented with a few flocks and found it effective*. *(…) He is my wife’s uncle*.*” (Farmer 11)*

### Influence of the poultry supply chain on reducing antibiotic usage

Three different supply chain models were identified among the participants, with different influences on changing practices.

In the traditional system, economic factors were identified by farmers as a barrier to reducing ABU. Indeed, antibiotics were considered a safe way to maintain productivity. High market fluctuations in output and feed prices were a barrier for farmers to change their practices; this was confirmed by drug sellers and traders. Feed was considered the highest expenditure and costs have increased since the pandemic. Moreover, chicken prices fluctuated constantly. Between December 2021 and February 2022, the price for one kilogram of chicken increased by 50%. This was explained by the Covid situation in December, which considerably lowered prices and the approach of the Têt festival in February when consumption increased. But after these particular events, prices decreased again, as reported by the respondents. During difficult times, some farmers reported producing at a loss and it was also common for farmers to obtain loans from drug sellers and pay them back later. Drug sellers make a profit on antibiotics, but according to them, they would have higher income if farmers used fewer antibiotics. Indeed, this would mean that chickens would be healthier, so farmers would be wealthier and could pay them back. Poor market conditions have also affected the number of farms. Indeed, some people have stopped raising chickens and this in turn has had a negative impact on drug sellers. Certain farmers solved their economic difficulties by combining several jobs, to increase their resilience (the multiple sources of income helped them to absorb shocks).

‘*They look beautiful*, *a good crest and shiny feathers’ (Trader 1)*

The conventional selling of chickens at the traditional market was considered a barrier to changing practices. Most of the chickens were bought by traders, through middlemen, to be sold on the wholesale market, in Bac Giang, and then distributed to Hanoi or Bac Ninh provinces. Traders did not ask farmers about their ABU because it was not a market requirement, no control was done at this level and farmers did not usually tell them the truth. According to traders, consumers did not care about this issue and bought chickens only for their taste. Farmers stated that producing quality chickens (tasty and safe) was one of the main motivations to reduce ABU. However, farmers reported that quality chickens were not sold at a higher price on the market, even though their production cost was higher.

“*They don’t care about this issue much*. *Traders just come to check our chicken flocks*. *They care about the chickens’ feathers and weight*. *They will be ok if the chickens have silky feathers and the same weight*. *If chickens are ugly*, *the feathers are shabby and sticky*, *traders will know that we use lots of medicines*.*” (Farmer 4)*

“*I’m always sure about the quality of my products*. *However*, *no one has requested quality products*. *Now*, *chickens are sold on the free market*, *so the prices are not different between farms*. *With the good quality of my chickens*, *the price should be higher but it’s not [the case] in the free market*.*” (Farmer 3)*

Integrated farms must comply with the integrator requirements and the integrator must comply with state policies. Antibiotics were therefore only used in the feed for the authorized time and the withdrawal time was applied strictly. These products were sold in supermarkets under the VietGAHP certificate that should include testing of residues. One integrator also started a brand of herb-fed chickens to target a new domestic market. However, the market was still insufficient for these types of products as consumers were identified as customarily buying their meat at traditional markets. Independent farmers that had the VietGAHP standard were not able to sell their products in supermarkets. Indeed, one farmer reported a lack of consumer demand. He was therefore obliged to sell them on the traditional market, but as the higher production prices were not reflected in higher purchasing prices, the solution was not profitable for him.

“*It seems that all Ta Lo chickens cannot be sold in supermarkets because the price is too high*. *Supermarket chicken meat is soft and cheap*. *The price of supermarket chicken meat is only as much as the price of our living chickens*. *So*, *if we sell them in the supermarket*, *the price of our chickens will be too high*.*” (Farmer 16)*

A solution developed by farmers to overcome economic difficulties was to organize themselves into cooperatives. Among our respondents, four farmers were part of an existing cooperative (farmers 9, 11, 13, 17 on the Fig 2) and two farmers wanted to create a new one in the area (farmers 3,4 on the Fig 2). The existing cooperative was created in 2014 by farmer 17 and included 10 members. Half of the farmers had been feeding their chickens with local hand-made probiotics since 2018 as they had to be free-range and AB residue free. Inputs were purchased in common (AB from Drug Seller 2) and the cooperative supported farmers in the selling process. They had the VietGAHP certificate. Two governmental provinces supported the development of the cooperative. A second cooperative with 17 members was soon to be officially created with a focus on producing “organic” chickens with less AB the application of new technologies. They were still to be fed with the probiotics and corn, but they were to stop using AB for the last 2 months. Feed were to be processed in common and the leader aimed to develop processed meat. A new collaboration to sell chickens to a shop specialized in quality chickens in Hanoi was under development. The trader responsible for the sale would be associated with the cooperative and would also provide them with drugs. This trader started this business to produce “clean food, drug-free chickens” (Trader 2) after realizing that most chicken meats contained antibiotics. According to one member, this cooperation would guarantee a better valorization of their products (higher selling price). Chickens were to be sold under the brand of the cooperative. The leader also intended to be part of the “One Commune One Product” program, supported by the government and aiming to promote high-quality products from a commune. This was a way for producers to better valorize their products by increasing consumer trust.

According to the respondents, the advantages of being part of a cooperative were the lower prices of the inputs (feed, drugs, DOCs), the assurance of having stable outputs, the possibility to share their technical expertise, and it’s legal existence. Moreover, they received technical and financial support from the government. Farmers involved in a cooperative didn’t mentioned barriers to being part of a cooperative. But they explained that other farmers did not want to join due to a lack of awareness.

“*Joining cooperatives is completely voluntary*, *the members discuss and implement together*. *There is no pressure and it is not required for everybody to follow*. *This is what the new type of cooperatives is like*, *so farmers feel comfortable*. *When joining*, *I can see many advantages*.*” (Farmer 17)*

## Discussion

By conducting qualitative research using participatory approaches, our study identified chicken farmers who have successfully reduced their antibiotic usage. Technical, economic, and social factors were all identified as having either a positive or a negative influence on changing practices. We were able to describe these factors as our sampling included both farmers who had reduced their ABU and farmers who hadn’t. For family commercial farms that reduced their ABU, their main objective was to produce good quality chickens, in terms of taste and food safety, while ensuring sufficient profits. In most case, changes in practice resulted from a combination of the three factors. For the cooperative, we identified that the farmers’ association was a means to reduce farm expenditures while sharing technical methods (local-handmade probiotics) and knowledge. It allowed the development of a new commercial outlets with higher profits for farmers. Other family commercial farms successfully reduced their ABU by improving their knowledge, biosecurity, and farming practices. We observed that integrated farms did not belong to the same social network as the independent family commercial farms. Their reduced ABU was mainly motivated by the integrator’s decision to comply with national regulations and to adhere to international market trends. The household farm used AB only for treatment as they farmed very few chickens and these were solely for their own consumption and might not need to reduce their ABU.

Our study also shed light on the need to study ABR and ABU practices from a systemic perspective, rather than focusing on motivations and changes in practice solely among individuals alone. Knowledge deficit models have made a significant contribution in exploring drivers of change by linking a poor level of knowledge and awareness with high ABU [30]. However, in our study, most farmers were aware of ABR causes and consequences and of good biosecurity practice, but remained high AB users due to socio-economic constraints and didn’t consider this to be problematic. This means that good ABU practice doesn’t depend only on knowledge but rather on the socioeconomic issues in which farmers are embedded. Our study provided evidence of the need to focus on social, economic, political and cultural factors when addressing changes in practice, as reported by other social science studies [30].

However, our study had with certain limitations. Our sampling method was based on the selection of participants by the local governmental authorities, followed by the snowball sampling method for the second phase. Respondents were highly connected and this might have reduced the diversity of opinions. However, it also helped to further understand the network of stakeholders and how relationships can contribute to reducing antibiotic use. Moreover, more interviews could have been conducted to explore further cooperatives or other farmers that have reduced their ABU. We validated our data by using multiple sources of data information (triangulation of interviews, observation, and participatory tools), team discussion on the matrix coding, and feedback to the participants [38]. The sampling method might also have contributed to a gender bias with an underrepresentation of women in our study. It has been shown that globally women were usually in charge of feeding activities while men were still in charge of the decisions in family poultry production [39]. This seems to be confirmed by our study, and would explain the higher proportion of men. Other explanations, might come from the employment area. During our survey, most of the women were not at home as they were working in industrial zones. A review conducted in South East Asia has shown that there was a gender difference in antibiotic usage [40], this was also raised by one of the drug sellers that we interviewed. Women might therefore have expressed different opinions on the topic. This once again underlines the need to conduct more studies on gender differences in antibiotic use in Vietnam [40].

The adoption of technical solutions was related to socio-economic factors. Farmers used local handmade probiotics because they were members of a cooperative that used them and because it was required to meet the quality standard requirements for downstream marketing. In addition, this helped to reduce farm expenditures, a particularly important concern for family commercial farms. Herbs were also used by one integrator to reach a new domestic market and to approach international markets. Applying high biosecurity in integrated farms was the results of a top-down decision from integrators, also to meet international markets. And finally, the increase of the usage of vaccines was a combination of higher disease pressure, the influence of veterinarians and other farmers, and the need to reduce farm expenditures. Technical solutions have been proven to effectively decrease ABU at farm level [41]. However, using these tools to change practices requires a combination of different factors that are highly dependent on political, economic, technical, and cultural factors. Thus, using more vaccines to decrease ABU would seem feasible in Vietnam because vaccines are accessible. However, in Madagascar, for example, vaccination in remote areas is compromised by difficult storage conditions and poor governmental support [42] making this solution less effective in this context. Poor biosecurity in family commercial farms was related to the economic pressure on farmers who were reluctant to take the risk of increasing the fallowing period, reducing density or applying other measures. However, the development of model farms that apply Enhanced Biosecurity Practices (EBP), through the support of FAO in collaboration with the Department of Livestock Production (DLP), has shown positive results. Indeed, these model farms use less AB and show higher profits than control farms [43]. Our study highlights the need to improve biosecurity in family commercial farms by demonstrating its economic benefits for farmers in Vietnam.

Farmers’ social networks were identified as playing a key role in knowledge and practices around ABU and ABR diffusion, and in initiating a change in practices. This information was gained from veterinarians, professors, drug companies, and governmental authorities through the organization of many workshops in the area. Farmers also relied on the experience of other farmers similarly to another study conducted in Vietnam [24]. Moreover, being part of the cooperative was also a consequence of social interactions. Indeed, farmers joined the cooperative because they were related to the cooperative leader or because they observed positive technical and economic results for other members. We also identified a drug seller who was particularly influential in promoting good ABU practice. Famers trusted him because he was perceived as having a lot of experience and because he was part of the SubDAHLP staff. Another study also highlights the greater confidence of farmers in official veterinarians [44]. Drug sellers have been identified in many studies as playing a key role in changing practices because they are the main source of advice for farmers [45]. Building trusted relationship between farmers and drug sellers appeared to be an important lever for the adoption of new solutions and the progressive changing of practices. But, in our study, similarly to farmers, drug sellers had varying levels of knowledge, influence and interest in reducing ABU and their motivations were not homogenous. The drug seller’s business model relies on their sales of antibiotics, feed additives, vaccines and, for some shops, feed or other goods, which make them highly dependent on farmers’ profitability. Thus, bad sanitary conditions have a socioeconomic impact on both farmers and drug sellers. Creating a less precarious business model for veterinarians could be a way to reduce ABU,—a question also raised in Europe—with a better remuneration of advice and the decoupling of antibiotic prescriptions and sales [46]. Some drug sellers did not, therefore, have a positive influence on the change of practice. This has already been observed in a previous study [44]. It is particularly important to address this issue as it has been shown, in Vietnam, that providing good advice to farmers contributes to ABU reduction [27]. The farmer’s social network has been identified, in France, to be a significant contributor to changing practices, particularly through knowledge diffusion. Indeed, farmers are embedded in an environment with social interactions, in particular knowledge diffusion [33]; in pig farming this has contributed to the diffusion of innovations [32], and to changing farmers’ practices regarding the use of pesticides and new soil management in France [47].

Downstream stakeholders such as supermarkets or specialized selling channels were identified by respondents as promoting good ABU practices. Chickens sold in supermarkets must have the national VietGAHP certificate that requires, among others, AB-residue testing and recording of ABU. In this context, integrators performed random on-farm testing and farmers were aware that they should no longer use AB for 35 days prior to selling. In the case of the cooperative, the development of a collaboration with a trader that sells higher-quality chickens in Hanoi seemed promising in regards to reduced ABU. Farmers must comply with the retailer’s requirements, in exchange for which they make more profit. This shows that there are other forms of socio-economic organization to produce quality food while being sustainable. These initiatives must be supported to improve farming practices and reduce ABU among small holders. The downstream level has also been shown to be a key element, in France, in the development of AB-free labels in poultry production [33]. In the pig sector, also in France, retailers took the initiative of meeting societal demand and guaranteeing outputs for producers with economic benefits for farmers [48]. It is therefore important to look at the market structure and the organization of value chain when considering ABU issues and to support a sustainable transition [49, 50]. However, family commercial farms had trouble selling their produce to supermarkets, even with the VietGAHP certificate. This lack of outlets has also been reported in pig production in Vietnam [51]. So, most of the independent farmers were selling local breed chickens at the traditional market to comply with cultural preferences, as already reported [27]. But at the traditional market, our respondents reported no economic advantages in producing chickens with lower ABU, as there was no product traceability and only scarce control. Low levels of regulation in the food supply chain are common in low- and middle-income countries (LMICs) [52], including Vietnam [53], and this is a driver of ABR in LMICs [54]. Thus, residues can be found in meat, as reported by our respondents and other studies [55, 56] that can lead to negative consequences on human health [57]. One solution to reduce ABU could be to develop a more controlled traditional market with better valorization for chickens raised with lower ABU. Food safety programs have been developed in Vietnam, including the organization of training in the local market; this could be a way of raising awareness of ABR issues among traders and retailers [58]. Our study shows that forms of coordination between actors and commercial valorization other than the integrated system exist in LMICs and that it is also possible to develop quality products for domestic supply.

Our study brings interesting contributions to the “trajectory of change” concept already used to study ABU reduction transition pathways in livestock production [32, 33]. We obtained a similar category of factors that influence changes in process, but the content of each factor varied between case studies. This shows that factors that influence change are context-dependent and that studying them from a systemic perspective allowed us to identify levers to be activated. The development of the cooperative in our study can be investigated according to the multi-level perspectives theories [59]. Innovations are likely to appear at the micro-level (niche) due to disruption at the meso level (sociotechnical regime) caused by changes at the macro (sociotechnical landscape) level [59, 60]. In our study, changes at the meso level were the raising awareness of consumers regarding food safety, the need for farmers to better valorize their products, and changes in regulations. This has created the opportunity for the development of ‘niche’ innovation represented by this cooperative. To promote the development and sustainability of these cooperatives around antibiotic use reduction, adjustments at the meso level must be made by, for example, developing new regulations or market incentives. More studies are required on this type of innovation that should also be encouraged by the government, especially when they are, as is the case here, associated with a safer product as recommended by Lamine [34]. In the case of the transition toward organic farming and integrated pest management to reduce the use of pesticides, collective dynamics have been identified as one of the main conditions for change, due to support around sharing experiences, difficulties, and methods [34]. In another example in Austria, for the development of the local food supply chain, the cooperation of farmers helped to reduce the cost of production, processing, and distribution, and sharing of experience [61]. It has also been shown that during their transition toward sustainable agriculture farmers rely on their social network where they find the support and the motivation to change [62].

The transition process in our study was progressive, as explained elsewhere [32–34] rather than the consequences of a trigger event as it has been observed in other studies [63]. There was no linear rationale decision to gain financial benefits or to contribute to public health [29], but rather a combination of different factors and motivations that have contributed to the reduction. Our result emphasizes the need to study behavior change, not only from the perspective of individuals but by furthering our understanding of the system in which the change takes place [33, 35].

Several research gaps were identified in our study. It as has already been advocated [40], there is a need to conduct further research on women’s access to innovations, as they play a key role in chicken production and, by extension, in the fight against ABR. Better involvement of this part of the population is required to ensure a sustainable change in practice. Moreover, neither consumers nor supermarkets were included in our survey. It would be interesting to study the consumers’ point of view on ABU reduction in depth. New levers could then be identified. Finally, we highlighted the importance of the bottom-up approach when studying changes in practice. Solutions must be co-developed by the relevant stakeholders at the local level through a co-building process to tailor them to the context and to enhance their effectiveness.

## Conclusion

At a time when many policy changes are being implemented in Vietnam, with increasingly strict regulations and a shift in livestock production from small-scale to more integrated systems, farmers are facing many changes in their production practices. Vietnamese farmers have been criticized for not caring and not engaging in ABU reduction and taking action to tackle ABR. However, at the local level, certain private initiatives are leading to a progressive change in practices. The development of farmers’ cooperatives, the influence of well-trained veterinarians and extension services projects have been successful in getting farmers to understand the need to reduce their ABU to improve food quality, reduce their farm expenditures, and in some cases, reduce the ABR burden. We believe that incorporating local innovations in the ABR stewardship program will contribute significantly to achieving the goal of ABU reduction in livestock production in Vietnam.

## Supporting information

S1 File Inclusivity in global research. (PDF)

S2 File Semi-structured interview guidelines for farmers and drug sellers, 2021–2022, Phu Binh district, Thai Nguyen province, Vietnam. (PDF)

S1 Table Farmer’s characteristics according to semi-structured interviews from December 2021 (phase 1) and February 2022 (phase 2) in Phu Binh district, Thai Nguyen province, Vietnam. AB: antibiotics; ASF: African Swine Fever, VietGAHP: Vietnamese Good Animal Husbandry Practices Factors (+): motivations to reducing AB; Factors (-): barriers to reducing AB, (?): unclear.
(PDF)

S2 Table Ranking of methods to reducing ABU by farmers and drug sellers from semi-structured interviews, phase 1, December 2021, Phu Binh district, Thai Nguyen province, Vietnam. NA: not addressed.
(PDF)

S3 Table Results of the proportional pilling of farm expenditure at the time of interviews and 5 years ago from semi-structured interviews, phase 1, December 2021, Phu Binh district, Thai Nguyen province, Vietnam. NA: not addressed.
(PDF)
