# Supplementary material for: Reducing antimicrobial use in chicken production in Vietnam: Exploring the systemic dimension of change
Source: PLoS One. 2023 Sep 8;18(9):e0290296. doi: 10.1371/journal.pone.0290296 (PMC10490891; doi:10.1371/journal.pone.0290296)
Supplement: S1 File — (PDF) [file pone.0290296.s001.pdf]

Inclusivity in global research

Ethical considerations, permits and authorship

This section is applicable to all research types.

Provide details as to who granted permissions and/or consent for the study to take place in the Methods section of your manuscript. This should include the names of **all** ethics boards, governmental organizations, community leaders or other bodies that provided approval for the study. If individuals provided approval refer to these people by their role or title but do not list their name(s).

Reported on page number: 9<br>This study was approved by the Ethics Review Board for biomedical research of Hanoi University of Public Health with the application number 021-391/DD-YTCC. The permit to conduct this work was given by the Sub-department of Animal Health and Livestock Production (SubDAHLP) of Thai Nguyen province.

If there were any deviations from the study protocol after approval was obtained please provide details of these changes in the Methods section of your manuscript.

N/A

Did this study involve local collaborators that are residents of the country where the research was conducted or members of the community studied? If you do not have any authors from said communities, please provide an explanation for this below.

The study included local collaborators that are Vietnamese residents where the study was conducted. They are co-authors of this work. Four co-authors are working in universities in Hanoi et one co-author is working in Thai Nguyen university. This study also involved students from Thai Nguyen university and local authorities.

Everyone listed as an author should meet PLOS' criteria for authorship and all individuals who meet these criteria should be included in the author byline, rather than the acknowledgements. Authorship criteria is based on the International Committee of Medical Journal Editors (ICMJE) Uniform Requirements for Manuscripts Submitted to Biomedical Journals - for further information please see here: https://journals.plos.org/plosone/s/authorship.

Human subjects research (e.g. health research, medical research, cross-cultural psychology)

Did you obtain written informed consent from a representative of the local community or region before the research took place? How did you establish who speaks for the community? Details of written informed

consent obtained from study participants should be reported separately in the Methods section of your manuscript.

Informed written consent from all respondents was obtained prior each interview after the study's objectives were explained to the participants by the interviewers. Explanations on the study's objectives and written consent were done in Vietnamese by one member of the team.<br>This information is provided in the Method section at page 8.

How did members of the local community provide input on the aims of the research investigation, its methodology, and its anticipated outcome(s)?

The study's area was selected based on the discussion with local collaborators (co-authors) and the Sub-department of Animal Health and Livestock Production (SubDAHLP) of Thai Nguyen province. The methodology to select the participants was defined with the local authorities of the selected district.

When engaging with the local community, how did you ensure that the informed consent documents and other materials could be understood by local stakeholders?

The informed consent was translated in Vietnamese and explained in Vietnamese to the participants. It was approved by the Ethic Committee of Hanoi Public Health University. The interview was conducted in Vietnamese.

Will the findings of the research be made available in an understandable format to stakeholders in the community where the study was conducted (e.g. via a presentation, summary report, copies of publications, etc.)? Please provide details of how this will be achieved.

A restitution meeting was organized in February 2023 to present the results to the participants. All the participants were invited to the restitution meeting. The presentation was done in Vietnamese and was followed by a discussion. A document with the slides of the presentation was distributed to the participants.

Non-human subjects research using specimens/ animals collected as part of the study, or those housed in archival collections. Examples include archaeology, paleontology, botany and zoology.

Did the permission you obtained from a local authority to perform the study include an agreement on access to outputs and benefit sharing? This may include procedures to enable fair distribution of the benefits and

N/A

resources arising from the research performed. Please include any details of Prior Informed Consent and Benefit Sharing Agreements obtained. These may be required by field-specific regulations, for example the Convention on Biological Diversity (CBD) and the associated Nagoya Protocol.

If the material used in your study was imported, please A) provide the year it was imported and B) indicate whether permits were obtained to import/export the materials used, C) provide details of any permits obtained. If this information is not available, please indicate this.

N/A

If you used archival specimens, please state how the material used in your study was acquired by the institute it is held in and provide details of any permits obtained for the original excavations/ sample collection. If this information is not available, please indicate this.

N/A

How was the potential cultural significance of the materials collected in your study to local communities considered in your research design? Were Indigenous peoples and/or local researchers and institutions involved with archaeological excavations / collection of specimens? If so, please provide a description of their involvement.

N/A

If your manuscript includes photographs of human remains please indicate whether authors obtained permission from descendants or affiliated cultural communities to do so.

N/A
