# Supplementary material for: Reducing antimicrobial use in chicken production in Vietnam: Exploring the systemic dimension of change
Source: PLoS One. 2023 Sep 8;18(9):e0290296. doi: 10.1371/journal.pone.0290296 (PMC10490891; doi:10.1371/journal.pone.0290296)
Supplement: S2 File — (PDF) [file pone.0290296.s002.pdf]

S2

File. Semi-structured interview guidelines for farmers and drug sellers, 2021-2022, Phu Binh district, Thai Nguyen province, Vietnam.

Interview guide – Farmers

Farm context

Trajectory and experiences:

Could you tell me your story on how you became a farmer? When did you start to work as a farmer and why? Did you work for another farm before working on this one?

Could you explain to me your experience on this farm? When did you start to work on this farm? Can you tell me how this farm has evolved over time? What have been the major changes in your business model (product price, flock size, source of drugs, …)?

What is the significant event that you have faced up until now?

Draw a timeline of the farm history and add the business model changes

Value chain:

Could you explain your daily activities at the farm? Who is working on the farm? Where do you buy day-old chicks (or pullet) and the feed? Where do you buy the drugs? To whom and where do you sell your products?

Draw value chain

Disease management

What do you do when you are facing chicken illness on your farm? What do you do to keep your chickens healthy? When you have these symptoms, what do you do to treat your chickens?

Change of practices

Antibiotic (opinion, usage, evolution of the usage)

Can you tell me your opinion about antibiotics in chicken production? Can you tell me your opinion on antibiotic resistance?

When was the last time you used antibiotics? For what? How often do you use them? For which purpose do you use them? Do you use antibiotics in prevention? What dosage do you use?

Do you ask advice for professional/other advice when you use an antibiotic?

Compared with when you started your activity, how your antibiotic use has evolved? Could you explain to me the story of this evolution and the most important actors?

Add the actors to the timeline

What do you think is the most reasonable way to use antibiotics?

Reducing antibiotic usage

What did you do to reduce your antibiotic usage? How did you reduce your antibiotic use? Biosecurity/density/alternative product/vaccination/…

Do you use herbs? Do you use pre-biotics or pro-biotics, or essential oils? Could you explain me when did you start to use these products and why?

Put on cards the identified methods and ask the respondent to classify them between the most useful, the most difficult, the most costly, the most sustainable...

Could you evaluate the evolution of the mortality of your flock before and after the reduction?

Motivations

What was your motivation to change your practices (at the start and now)? Did these changes meet your expectations, do you know why?

Barriers

What are the main difficulties that you are facing on your farm? What is the difficulty that you are facing since you have reduced your antibiotic usage? What is the negative impact of this change of practices? On economic, animal welfare, and time spent on the farm. Who is the most impacted by these difficulties on your farm?

Levers

What are the positive impacts that these changes had on your farm activity? On economic, animal welfare, recognition by other farmers, personal well-being… Who is the most impacted by these changes?

What is the link between the evolution of your business model and the reduction of the antibiotic?

Do you advocate around you the need of using less Ab? To whom? Why?

Evolution of the expenditure items: proportional pilling

Could you enumerate the different expenditure items that you have on your farm?

Draw circle on a paper that represents the different expenditure items

Please indicate the relative importance of the expenditure items in terms of expenditure before reducing antibiotic usage.

Place the beans (100) on the sheet and ask the participant to place them in the different circles
Note how many stones are in each circle

Now, we ask you to do the same thing but after that, you have reduced your antibiotic use.

Replace the beans (100) on the sheet and ask the participant to place them in the different circles
Note how many stones are in each circle

Interactions

With drug sellers, veterinarians, ...

Where do you buy your antibiotics? Where do you buy your alternative products? Does a vet is coming to treat your animal? Besides treatment, what do you discuss with the vet?

What is the opinion of the vet on the fact that you have reduced your antibiotic use? How do they support (or not) you?

Do you have someone whom that help you to reduce your antibiotic usage on your farm? Could you explain to me who is it and what this person is doing?

Farmers

What relation do you have with other farmers? Do you have other farmers around you that have changed practice? Do you share with them your antibiotic usage practice?

Others

With whom else do you interact (chicken company, traders, customers, …)? Could you explain what type of relationship you have with them?

Conclusion

For you, what is a good farmer today?

Is there anything you want to add?

Do you think we've approached everything relevant?

Do you have any questions?

Thank you

Interview guide – Veterinarians/Drug sellers

Working context

Trajectory and experiences:

Could you tell me your story on how you became a vet/drug seller? When did you start to work as a vet and why? Where did you start, did you work for different kinds of practices and sectors in your career, etc.

Could you explain to me your experience as a vet? When did you start to work in this company/agency/…? Are you the owner? Can you tell me how your status has evolved over time? What is the significant event that you have faced up until now?

What have been the major changes in your business model?

Draw a timeline of the history of the drug agency and add the evolution of the business model

Practice characteristics:

Could you explain your daily activities as a vet? Who is working with you? Where do you buy drugs/herbs/alternative products…? To whom and where do you sell your products?

Are you doing farming visits? What is your clientele?

Could you describe your activities in the shop?

Draw the value chain of the antibiotics

Disease management

What do you do when you are faced with chickens' illness on the farm? Do you perform an autopsy or another test? Do you give antibiotics?

Do you give advice to the farmers besides the treatment of their chickens (biosecurity, feed, …)?

Business model

Antibiotic (opinion, usage, evolution of the usage)

Can you tell me your opinion about antibiotics in chicken production? Can you tell me your opinion on antibiotic resistance?

Do you use antibiotics now in your business work? When was the last time you used antibiotics on a farm? For what? How often do you use them? For which purpose do you use them?

What do you think is the most reasonable way to use antibiotics?

What dosage of antibiotics do you use (compared to manufacturer instruction)? Do you recommend using antibiotics for prevention on the farm? If yes for which purpose?

Compared with when you started your activity, how your antibiotic use has evolved? Could you explain to me the story of this evolution and the actors involved?

Draw a timeline

Reducing antibiotic usage

What did you do to reduce your antibiotic usage on the farm? How did you reduce your antibiotic use? Biosecurity/density/alternative product/vaccination/…

Do you use herbs? Do you use pre-biotics or pro-biotics, or essential oils? Could you explain me when did you start to use these products and why? Where do you buy this product?

Put on cards the identified methods and ask the respondent to classify them between the most useful, the most difficult, the most costly, the most sustainable...

Motivations

What were your motivations to change your practices (at the start and now)? Did these changes meet your expectations, if not do you know why?

Did you receive any training to change your practices, if yes from whom?

Did you have a mandatory requirement from your company? Do you reduce antibiotic use to comply with the new regulations?

Do you think that you have to advocate this change of practices to other vets, farmers, …? How do you see your position in fighting against antibiotic use?

Barriers

What is the difficulty that you are facing since you have reduced your antibiotic usage? What is the negative impact of this change of practices? On economic, animal welfare, …?

What is the positive impact that these changes had on your vet activity? Was it the farmers that ask to help them? Do you think that reducing antibiotics will change the farm systems?

Levers

What are the positive impacts that these changes had on your activity? On economics, animal welfare, recognition by farmers, another vet, opinion… Who is the most impacted by these changes?

Do you advocate around you the need of using less AB? To whom? Why?

Evolution of the expenditure items: proportional pilling

Could you enumerate the different expenditure items that you have?

Draw circle on a paper that represents the different expenditure items

Please indicate the relative importance of the expenditure items in terms of expenditure before reducing antibiotic usage.

Place the stones (100) on the sheet and ask the participant to place them in the different circles
Note how many stones are in each circle

Interactions

Farmers

What are the characteristics of the farmers that are asking to reduce their antibiotic use? What do you do to help them? What are their motivations to reduce their usage?

Vets

What relation do you have with other vets? Do you share with them your antibiotic usage practice? Do you sometimes disagree?

Others

With whom else do you interact (chicken company, traders, customers, …)? Could you explain what type of relationship you have with them?

Conclusion

For you, what is a good vet today? When you entered the profession?

Is there anything you want to add?

Do you think we've approached everything relevant?

Do you have any questions?

Thank you
