# Supplementary material for: Reducing antimicrobial use in chicken production in Vietnam: Exploring the systemic dimension of change
Source: PLoS One. 2023 Sep 8;18(9):e0290296. doi: 10.1371/journal.pone.0290296 (PMC10490891; doi:10.1371/journal.pone.0290296)
Supplement: S1 Table — AB: antibiotics; ASF: African Swine Fever, VietGAHP: Vietnamese Good Animal Husbandry Practices Factors (+): motivations to reducing AB; Factors (-): barriers to reducing AB, (?): unclear. (PDF) [file pone.0290296.s003.pdf]

S3

Table. Farmer's characteristics according to semi-structured interviews from December 2021 (phase 1) and February 2022 (phase 2) in Phu Binh district, Thai Nguyen province, Vietnam.

AB: antibiotics; ASF: African Swine Fever, VietGAHP: Vietnamese Good Animal Husbandry Practices Factors (+): motivations to reducing AB; Factors (-): barriers to reducing AB. (?): unclear.

Farmer (phase of interview)	Gender, age, education	Years of experience	Date of installation	Production system, number/flock, type, housing, number of workers	Description and farm evolution	ABU profile	ABU practice	Factors (+)	Factors (-)	Methods
Farmer 0 (pilot)	Male, 48, primary school	15	2008	Family commercial, 15000, DOCs, semi-confined, 1	High profit motivation, started to work on his own in 2017, increased farm size	Have reduced	Prevention (periodically throughout the animal's life) and treatment	Chicken quality, food safety, lower farm expenditure	Knowledge, breeding condition (weather)	Better vaccination, better farming practices workshops, increased knowledge
Farmer 1 (1)	Male, 25, university	3	2021	Contract, 15000, meat, confined, 4	Farm manager, change of job in 2021 for a new company because they were overusing AB, main activity	Have reduced	Prevention (first week) and treatment	Chicken quality, food safety, requirements of integrator	Lack of output	Herbs in feed, chicken care, quality chicken brand (marketing), biosecurity, change of job
Farmer 2 (1)	Male, 32, university	5	2021	Contract, 40000, meat, confined, 4	Farm manager, owner of another farm of the same chicken company, main activity, previous job at the veterinarian faculty	Have reduced	Prevention (first week + feed with AB until 15d) and treatment until 35 d	Requirements of the integrator (Motivation of the integrator: productivity, profit, to maintain its reputation)	Lack of output	Biosecurity, regulations
Farmer 3 (1)	Male, 45, college	20	2015	Family commercial, 4000, meat, semi-confined, 2 (family)	Farm owner, focus only on chickens, increased farm size in 2019, previous job at the veterinary station, bought another farm in 2016, work with his wife	In the process of reducing, same amount	Prevention (rarely, young) and treatment	Avoid antibiotic resistance, lower farm expenditure	Infectious pressure, density	Improved biosecurity (biomatress), want to develop a cooperative, vaccination, workshops, main activity (focus only on that)

Farmer 4 (1)	Male, 47, highschool	15	2007	Family commercial, 3000, meat, semi-confined, 1	Increase farm size over the time, no husbandry training, saw profit of farming and continued, pigs, dogs	In the process of reducing, increased amount	Prevention (rarely, change of season) and treatment	Lower farm expenditure, chicken quality	Intensification, high density	Herbs (2019), want to develop a cooperative, want to reduce density, garlic
Farmer 5 (1)	Male, 44, highschool	14	2016	Family commercial, 3000, meat, semi-confined, 1	High profit, increased farm 5 years ago	Increased	Prevention (periodically throughout the animal's life) and treatment		Infectious pressure (multiple diseases, density), economic difficulties, no network of advice	
Farmer 6 (1)	Male, 36, highschool	12	2008	Family commercial, 2000, meat, semi-confined, 1	Invest in the farm in 2018, stopped raising pigs because of ASF, chickens (main income), crops, no training on farming, start because everyone was raising chickens	In the process of reducing (?), same amount	Prevention (periodically throughout the animal's life) and treatment		Infectious pressure (density)	Fallowing period > 5 months
Farmer 7 (1)	Female, 45, highschool	12	2013	Family commercial, 1500, meat and eggs, semi-confined, 1	Depending on the market conditions switch between laying hens and chicken meat, continue breeding because of the benefit, also drug seller, few training on livestock from Woman's Union	In the process of reducing (?), increased	Prevention (?) and treatment		Infectious pressure (more disease), lack of alternatives to AB	
Farmer 8 (1)	Male, 42, college	14	2018	Family commercial, 6000, DOCs, semi-confined, 2 (family)	Increased size over the time, bought 2 incubators in 2018, only source of income, stop raising pigs (too much work), took over family farms	Same amount	Prevention (periodically throughout the animal's life) and treatment		Don't know how to reduce, high density, downtime, lack of alternatives to AB	
Farmer 9 (1)	Male, 48, highschool	16	2016	Family commercial, 5000, meat, semi-confined, 2 (family)	Work in husbandry (main job) and reforestation, no training on husbandry, studied medicine, member of the cooperative	Have reduced	Prevention (periodically throughout the animal's life) and treatment	Lower farm expenditures, chicken quality, safer products, maximize productivity (awareness of animal husbandry)	More complicated, time-consuming	Local hand-made probiotics (2020), cooperative

Farmer 10 (1)	Female, 52, secondary school	32	1989	Household, 100, meat, eggs, DOCs, free range, 3 (family)	Increased number of chickens in 2016 but too many diseases so reduced the number, 3 pers (family)	Same amount	Treatment	/	/	Small-scale AB only for treatment
Farmer 11 (2)	Male, 42, highschool	13	2021	Family commercial, 1000+1200, meat and eggs, semi-confined	Main job, was working at his father-in-law farms, built his own farm in 2021, member of the cooperative	Have reduced	Prevention (change of season) and treatment	Lower farm expenditures	More complicated, time-consuming	Local hand-made probiotics (2020), cooperative
Farmer 12 (2)	Female, 41, secondary school	19	2012	Family commercial, 1000, meat, semi-confined, 2 (family)	Was raising at her father-in-law place	In the process of reducing (?), same amount	Prevention (first week), treatment		Infectious pressure, (« pollution » of the land)	Workshops on vaccination, better vaccination
Farmer 13 (2)	Male, 37, secondary school	13	2009	Family commercial, 4000, meat, semi-confined, 2 (family)	Took over family farms, main activity, member of the cooperative since 2016, increased in size over the time, used industrial feed in the past	Have reduced	Prevention (change of season) and treatment	Chicken quality, lower farm expenditures	Workload	Local hand-made probiotics, improved feed quality, cooperative, specialized selling channel
Farmer 14 (2)	Male, 54, primary school	5	2017	Contract, 8000, meat, confined, 1	Contacted by a representant of the company, main activity, has also her own flock, training by technician of the company, started because she saw that it was profitable	Have reduced (don't want to reduce)	Prevention (first week + feed with AB until 15d) and treatment until 35 days	Requirements from the integrator, random on-farm control	Infectious pressure	Regulations
Farmer 15 (2)	Male, 56, secondary school	22	2000	Family commercial, 1800, meat, semi-confined	Stopped raising pigs, expanded the farm in 2011, buffaloes	Have reduced	Treatment	Better productivity		Biosecurity (hygiene, water, feed), better farming practices, knowledge
Farmer 16 (2)	Male, 38, highschool	20	2002	Family commercial, 7000, meat, semi-confined, 2 (family)	Automatization and increased size (2012), VietGAHP (2018), cooperative (2021), main activity, good activity to earn money	Increased	Prevention (?), treatment		Infectious pressure	
Farmer 17 (1,2)	Male, 58, college	20	2001	Family commercial, 4000, meat, semi-confined, 2 (family)	Leader of the cooperative created in 2014, new cooperative 2022, start probiotic in 2018, increased size of the flock in 2010 (rented a land) and reduced	Have reduced	Treatment	Reduce ABU, chicken quality, better profit, entrepreneurial motivations, valorization of		Local hand-made probiotic, cooperative, specialized selling channel

flock size in 2018	products of his hometown, better benefit for farmers, production of safe products
