# Supplementary material for: Reducing antimicrobial use in chicken production in Vietnam: Exploring the systemic dimension of change
Source: PLoS One. 2023 Sep 8;18(9):e0290296. doi: 10.1371/journal.pone.0290296 (PMC10490891; doi:10.1371/journal.pone.0290296)
Supplement: S2 Table — NA: not addressed. (PDF) [file pone.0290296.s004.pdf]

# S4

**Table: Ranking of methods to reducing ABU by farmers and drug sellers from semi-structured interviews, phase 1, December 2021, Phu Binh district, Thai Nguyen province, Vietnam.** NA: not addressed.

| | Effectiveness | Feasibility |
|---|---|---|
| Farmer 0 | NA | NA |
| Farmer 1 | 1. Herbs in the feed<br>2. Hygiene<br>3. Human ressources<br>4. Vitamins | NA |
| Farmer 2 | 1. Vaccine<br>2. Hygiene<br>3. Farm environment<br>4. Alternative feed additives | 1. Vaccine<br>1. Alternative feed additives<br>2. Hygiene<br>2. Farm environment |
| Farmer 3 | 1. Vaccine<br>2. Spray antiseptic<br>2. Bio mattress<br>3. Alternative feed additives | NA |
| Farmer 4 | 1. Sanitizer<br>2. Isolation<br>3. Vaccine<br>4. Herbs<br>5. Farm environment<br>6. AB in water<br>7. AB injection | 1. Farm environment<br>2. Herbs<br>3. Isolation<br>4. Vaccination<br>5. AB in water<br>6. AB injection<br>7. Sanitizer |
| Farmer 5 | 1. Hygiene<br>2. Prebiotic<br>3. Probiotic<br>4. Herbs | 1. Hygiene<br>2. Prebiotic<br>3. Probiotic<br>4. Herbs |
| Farmer 6 | 1. Density<br>2. Monitoring<br>3. Hygiene<br>3. Sanitation<br>4. Herbs<br>5. Probiotics | 1. Hygiene<br>1. Sanitation<br>2. Herbs<br>2. Probiotics<br>3. Density<br>4. Monitoring |
| Farmer 7 | 1. Prebiotic<br>2. Vaccine<br>3. Temperature<br>4. Honey | NA |
| Farmer 8 | 1. Sanitizer<br>2. Clean feed and water<br>3. Alternative feed additives | 1. Alternative feed additives<br>2. Clean feed and water<br>3. Sanitizer |
| Farmer 9 | NA | NA |
| Farmer 10 | NA | NA |

| | | |
|---|---|---|
| Drug seller 1 | 1. Hygiene<br>1. Alternative feed additives | 1. Hygiene<br>2. Alternative feed additives |
| Drug seller 2 | NA | NA |
| Drug seller 3 | 1. Vaccine<br>2. Clean feed<br>2. Spray antiseptic<br>3. Probiotic | 1. Vaccine<br>2. Clean feed<br>2. Spray antiseptic<br>3. Probiotic |
| Drug seller 4 | Not included | Not included |
| Drug seller 5 | NA | NA |
| Drug seller 6 | 1. Vaccine<br>2. Garlic<br>3. Tea<br>4. Probiotic<br>5. Tonic<br>6. Honey<br>7. Herbs<br>8. Vitamins<br>8. Leaf | 1. Garlic<br>2. Vaccine<br>3. Tea<br>4. Probiotic<br>5. Tonic<br>6. Honey<br>7. Herbs<br>8. Vitamins<br>8. Leaf |
| Drug seller 7 | 1. Vaccine<br>2. Breed<br>2. Vitamins<br>3. Sanitation<br>3. Clean feed<br>4. Spray<br>4. Feed quality<br>4. Batch | 1. Vaccine<br>2. Breed<br>3. Clean feed<br>3. Sanitation<br>3. Vitamins<br>4. Spray<br>4. Feed quality<br>4. Batch |
| Drug seller 8 | 1. Vaccine<br>2. Vitamins<br>3. Liver detox.<br>3. Hygiene spray<br>4. Probiotic | 1. Vaccine<br>2. Vitamins<br>2. Probiotic<br>3. Liver detox.<br>4. Hygiene spray |
| Drug seller 9 | NA | NA |
| Drug seller 10 | 1. Vaccine<br>2. Hygiene<br>3. Density<br>4. Probiotic<br>4. Essential oil<br>4. Herbs<br>4. Vitamins<br>4. Garlic essential oil<br>4. Garlic | 1. Vaccine<br>2. Hygiene<br>3. Density<br>4. Vitamins<br>5. Probiotic<br>5. Essential oil<br>5. Herbs<br>5. Garlic essential oil<br>5. Garlic |
