# Supplementary material for: Reducing antimicrobial use in chicken production in Vietnam: Exploring the systemic dimension of change
Source: PLoS One. 2023 Sep 8;18(9):e0290296. doi: 10.1371/journal.pone.0290296 (PMC10490891; doi:10.1371/journal.pone.0290296)
Supplement: S3 Table — NA: not addressed. (PDF) [file pone.0290296.s005.pdf]

S5

Table: Results of the proportional pilling of farm expenditure at the time of interviews and 5 years ago from semi-structured interviews, phase 1, December 2021, Phu Binh district, Thai Nguyen province, Vietnam. NA: not addressed.

		Time of the interview	5 years ago
	Expenditure	% expenditure	% expenditure
Farmer 0	NA	NA	NA
Farmer 1	Antibiotic	3 (3%)	NA
	Vaccine	37 (37%)	
	Vitamins	29 (29%)	
	Herbs	31 (31%)	
Farmer 2	Feed	31 (31%)	NA
	Antibiotic	6 (6%)	
	Vaccine	13 (13%)	
	Alternative feed additives	13 (13%)	
	Hygiene	12 (12%)	
	Cost	25 (25%)	
Farmer 3	Feed	71 (71%)	67 (67%)
	Antibiotic	4 (4%)	13 (13%)
	Vaccine	5 (5%)	2 (2%)
	Alternative feed additives	6 (6%)	7 (7%)
	Bio mattress	9 (9%)	0 (0%)
	Breed	10 (10%)	11 (11%)
Farmer 4	Feed	40 (40%)	21 (38,2%)
	Antibiotic	10 (10%)	10 (18,2%)
	Vaccine	14 (14%)	13 (23,6%)
	Herbs	17 (17%)	0 (0%)
	Breed	19 (19%)	11 (20%)
Farmer 5	Feed	50 (50%)	31 (41,9%)
	Antibiotic	17 (17%)	10 (13,6)
	Vaccine	8 (8%)	8 (10,8%)
	Prebiotics	4 (4%)	3(4%)
	Probiotics	9 (9%)	6 (8,1%)
	Herbs	7 (7%)	8 (10,8)
	Breed	5 (5%)	8 (10,8%)
Farmer 6	Feed	37 (37%)	28 (35,5%)
	Antibiotic	18 (18%)	13 (16,5%)
	Vaccine	10 (10%)	7 (9,5%)
	Hygiene	5 (5%)	5 (6%)
	Herbs	13 (13%)	9 (11,5%)
	Breed	17 (17%)	17 (21%)
Farmer 7	NA	NA	NA
Farmer 8	Feed	47 (47%)	47 (47%)

	Antibiotic	18 (18%)	18 (18%)
	Vaccine	7 (7%)	7 (7%)
	Alternative feed additives	5 (5%)	5 (5%)
	Hygiene	5 (5%)	5 (5%)
	Breed	18 (18%)	18 (18%)
Farmer 9	Feed		
	Antibiotic	NA	NA
	Vaccines		
	Probiotics		
	Tonic		
	Breed		
	Infrastructure		
Farmer 10	NA	NA	NA
Drug seller 1	NA	NA	NA
Drug seller 2	Antibiotic	15 (15%)	15 (15%)
	Vaccine	36 (36%)	36 (36%)
	Probiotic	9 (9%)	9 (9%)
	Vitamin	24 (24%)	24 (24%)
	Sanitizing	16 (16%)	16 (16%)
Drug seller 3	Antibiotic	19 (19%)	19 (19%)
	Vaccine	28 (28%)	28 (28%)
	Probiotic	20 (20%)	20 (20%)
	Tonic	23 (23%)	23 (23%)
	Antiseptic	11 (11%)	11 (11%)
Drug seller 4	*Not included*		
Drug seller 5	Antibiotic	16 (16%)	30 (30%)
	Vaccine	33 (33%)	25 (25%)
	Alternative feed additives	36 (36%)	30 (30%)
	Antiseptic	15 (15%)	15 (15%)
Drug seller 6	Antibiotic	39 (39%)	58 (58%)
	Vaccine	16 (16%)	9 (9%)
	Probiotic	12 (12%)	12 (12%)
	Tonic	24 (24%)	14 (14%)
	Antiseptic	9 (9%)	7 (7%)
Drug seller 7	Antibiotic	16 (26,7%)	16 (26,7%)
	Vaccine	7 (11,7%)	7 (11,7%)
	Alternative feed additives	26 (43,3%)	26 (43,3%)
	Sanitizer	11 (18,3%)	11 (18,3%)
Drug seller 8	Antibiotic	23 (23%)	23 (23%)
	Vaccine	28 (28%)	28 (28%)
	Tonic	28 (28%)	28 (28%)
	Sanitizing	21 (21%)	21 (21%)
Drug seller 9		NA	NA
Drug seller 10		NA	NA
